# Interprofessional Educational Needs for Shared Governance of Integrated Care

**DOI:** 10.5334/ijic.7674

**Published:** 2024-05-06

**Authors:** Myonghwa Park, Eunjeong Choi, Miri Jeong, Hyun-Ju Seo, Jahyeon Kim, Eunkyung Seo

**Affiliations:** 1 College of Nursing, Chungnam National University, Daejeon, Republic of Korea; 2 Department of Public Health, Graduate School, Yonsei University, Seoul, Republic of Korea; 3 Department of nursing, Joongbu University, Geumsan-gun, Chungnam-do, Republic of Korea; 4 Chung-ang University Gwangmyeong Hospital, Gwangmyeong-si, Gyeonggi-do, Republic of Korea

**Keywords:** integrated health care systems, community health services, interprofessional education, patient-centered care, needs assessment

## Abstract

**Introduction:**

This study investigated the educational needs of integrated care among professionals in the public sector of healthcare and social care services in South Korea.

**Methods:**

A cross-sectional secondary data analysis was performed. Original data were obtained from 10 metropolitan communities with a convenience sample of 210 integrated care professionals. The Borich Needs Assessment Model and the Locus for Focus Model were used to examine the priority educational needs of each integrated care professional.

**Results:**

This study analyzed the key details of educational needs in integrated care by focusing on the competencies of integrated care approaches for person-centered care, interprofessional collaboration, and community involvement. The core educational needs of community care administrators, care coordinators, healthcare and social care providers, and community health champions, which are common to all professionals, and the specific educational needs for each type of professional were demonstrated, which contained specific content to implement integrated care.

**Conclusion:**

This study provides an opportunity to comprehensively understand the educational needs of integrated care professionals based on their competencies. They want better interprofessional cooperation through networking and collaborative strategies. The results of this study may be utilized as fundamental data by future instructors to provide evidence-based education programs.

## Introduction

The prevalence of chronic diseases and various healthcare and social care needs increase as the population ages, and integrated care is often suggested as a future healthcare model. Integrated care is acknowledged for its importance in prospects and global strategies for health-service delivery [1], and various integrated care models have been implemented in many countries [2,3,4]. The integrated care framework suggested by the World Health Organization [WHO) involves five interconnected approaches: empowering individuals and communities, enhancing governance and accountability, reshaping healthcare models, integrating services across sectors, and fostering a supportive environment [5].

Addressing people’s complex needs requires knowledge and skills from multiple disciplines in various fields [6]. Collaboration between healthcare and social service professionals is especially important to improve integration [7]. Therefore, professionals require knowledge, skills, and competencies in community settings to provide effective and high-quality integrated care. A previous study established that integrated care education should focus on teamwork, communication, role awareness, professional and personal development, practice development, leadership, and partnerships among professionals [8]. Other studies have emphasized the importance of context-driven learning [9]and training in relevant systems for integrated care [10]. The key competencies for integrated health services are patient advocacy, communication, teamwork, people-centered care, and continuous learning [11]. Moreover, for the integrated care of older adults, healthcare and nursing care professionals should understand the characteristics of the elderly and promote organic collaboration with others [12].

Interprofessional education takes place when students from multiple professions come together to learn from each other, fostering effective collaboration and enhancing health outcomes [13]. Several key challenges hinder the successful implementation of integrated care frameworks. First, there is a lack of a shared and comprehensive understanding of integrated care principles and practices among key stakeholders, including healthcare professionals, administrators, and policymakers [14]. This absence hindered effective collaboration and coordination. Additionally, the lack of a standardized guide for achieving integration poses a significant barrier [15]. Services that are duplicated or poorly coordinated can result in inefficient resource use and increased costs [16,17]. Therefore, promoting integrated care and enhancing interprofessional education are crucial for reducing costs and improving the overall quality of care. IPE (implemented to enhance integrated care) is often studied in the primary-care setting [18,19]. However, community-based IPE is relatively rarely conducted [20], and the participants in these studies have often been students [20,21,22,23] rather than professionals who work in the community.

Competency encompasses the specific knowledge, skills, abilities, and behavioral traits that an employee must possess and exhibit to perform their role effectively [24]. The world identifies five crucial competencies for successful integrated health services: patient advocacy, effective communication, teamwork, people-centered care, and continuous learning [5]. Defining individual roles and outlining the associated behaviors essential for successful role fulfillment within an integrated care system framework can enhance the effectiveness of professionals [25]. Therefore, identifying the educational needs of professionals based on the competencies of integrated care is valuable for implementing an effective integrated care model.

Although numerous models and examples of integrated care exist, sufficient exploration of how healthcare and social care professionals are prepared and trained in these settings is lacking [26,27]. Previous studies have investigated the educational needs for integrated care. However, most studies have focused on the educational needs of professionals who provide integrated care for patients with specific diseases or from different professional areas, such as psychiatric consultants or palliative care-link nurses [28,29,30]. Integrated care in a community setting is often delivered by multiple professionals, such as healthcare and social service providers, who work closely with community residents using a shared workflow. With the increased implementation of integrated care models, there is a need for a specific workforce of integrated care providers trained to deliver integrated care in new ways and meet the needs of community residents. Therefore, it is necessary to assess educational needs to design practical training programs to produce competent integrated care professionals who can provide integrated care to community residents. This study aimed to determine the educational needs of integrated care by the type of health and social care professional to identify the priorities of educational needs based on integrated care competencies.

## Research Methods

### Participants and Data Collection

This was a cross-sectional secondary data analysis of integrated care professionals in the community using an educational needs assessment survey. In the original study, the enrolled participants were integrated care professionals working in health and social care-related centers in 10 cities in South Korea that provided integrated care as a national pilot project. Convenience sampling is also performed. Professionals with less than 3 months of work experience were excluded. The researchers received permission from the representatives of health and social welfare centers via telephone and sent documents describing an overview of the research. The researchers sent the links to the electronic survey forms via email. Electronically informed consent was obtained from all participants. An online survey was conducted from December 2020 to January 2021, during which 210 respondents anonymously completed the survey. This convenience sample included integrated care professionals distributed across Korea and various practice settings. Only healthcare and social care professionals working in community settings were included.

Community-based integrated care professionals in South Korea collaborate to provide a range of services to meet the needs and circumstances of care recipients. The deployment of integrated care professionals in South Korea commenced in 2019. The roles of these professionals are still under development, with ongoing efforts to establish clear job descriptions, competencies, and training programs; however, research regarding this workforce remains limited. Administrators in local governments are responsible for the overall coordination of community-based integrated care, developing and implementing relevant policies, operating centers, and monitoring the performance of care providers. Care coordinators are frontline professionals, such as nurses and social workers, who work with individuals and their families to assess their unmet needs, develop care plans, and connect appropriate services in the community; health and social services providers implement direct health and social services when they are referred by care coordinators. Community health champions are peer supporters who share health messages with community residents in a friendly manner.

### Measurement Tools

A study was conducted to identify competencies for integrated care using practical experience in integrated care (23), and a list of competencies was used in this study (Appendix 1). The survey asked integrated care professionals to report their opinions on which integrated care competencies are essential for their functioning by asking what is important in their job, how often they perform, and how much they want to learn, to identify their specific learning needs in integrated care practice. Although they are equipped with primary care principles from training programs, learners often encounter clinical settings that hinder their application [31]. Questioning practitioners regarding their educational needs can establish a foundation for developing practical educational programs. To understand educational needs, it is necessary to consider both the importance and performance of specific competencies. Importance refers to the degree to which it meets the purpose of the competencies, and the performance of competencies refers to how well competencies are performed. The survey questionnaires were validated by researchers and experts (seven professors of medicine, nursing, public health, and social work; two nurse practitioners; and one social worker). The questionnaire comprised items rated on a 5-point Likert scale (1 = not at all important; 5 = very important), with higher scores indicating greater importance in integrated care practice. Participants’ performance levels regarding integrated care practice were measured on a 5-point Likert scale (1 = not at all performed; 5 = performed very much), with higher scores indicating better performance in integrated care practice.

### Data Analysis

SPSS software (version 22.0) was used to analyze the data. Frequency analysis was conducted to analyze the characteristics of the participants. An independent *t*-test was applied to the traditional importance-performance analysis method by Martilla and James (1977) [32] to determine the gap between integrated care professionals’ perceptions of the levels of importance and performance in integrated care practices. The Borich Needs Assessment Model [33] was used to identify the educational needs of integrated care professionals and their order of priority by considering the mean importance weight. To present a reference point for the order of priority, the Locus for Focus Model [34] was used to determine the number of items included in the high discrepancy, high importance (HH) quadrants. The Locus for Focus Model achieves this by plotting two key measures: the required change level, positioned on the horizontal axis, and the average difference between the required and perceived change levels, plotted on the vertical axis [34]. In this study, average performance served as the cutoff point on the horizontal axis, whereas the average difference between performance and importance was defined as the cutoff point on the vertical axis. HH was the first quadrant, and indicated the highest priority of education needs among the four quadrants; high discrepancy, low importance (HL) was the second quadrant and indicated a lower importance level than the average but a higher degree of discrepancy than the average. High-priority needs according to the Borich Needs Assessment Model were considered in the number of items, and when items from both methods corresponded, they were selected as the items with the highest priority. Items that were highly ranked in the Borich Needs Assessment Model, but were included in the HL quadrant, were selected as items of second priority.

### Ethical Consideration

This study was approved by the Institutional Review Board of Chungnam National University (No.202009-SB-121-01).

## Results

### Participants’ Characteristics

The demographic characteristics of the participants are presented in Table 1. They comprised 210 integrated care professionals working in integrated care facilities within the community. Most participants (66.4%) were female, with a mean age of 45.42 years and a mean length of work experience of 10.01 years. The education levels of the professionals were 61.4% with a bachelor’s degree and 22.8% with a master’s degree. Their positions were community care administrators (9.1%), care coordinators (34.9%), healthcare and social care service providers (29.5%), and community health champions (26.6%). Before participating in this study, over 70% of the professionals had not received any formal training or courses on integrated care.

**Table 1:** The demographic characteristics of participants.


	(N = 210)

CHARACTERISTIC	FREQUENCIES (%) OR MEAN (SD)

Sex	

Female	160 (66.4%)

Male	81 (33.6%)

Professionals’ highest degree	

≤College diploma	38 (15.8%)

Baccalaureate degree	148 (61.4%)

≥Master’s degree	55 (22.8%)

Job	

Community care administrators	22 (9.1%)

Care coordinators	84 (34.9%)

Health and social services providers	71 (29.5%)

Community health champions	64 (26.6%)

Experience of integrated care education	

Yes	69 (28.6%)

No	172 (71.4%)

Age	45.42 (SD = 10.50)

Years of experience	10.01 (SD = 8.82)


### Educational Needs in Core Integrated Care Competencies

**Figure 1:**
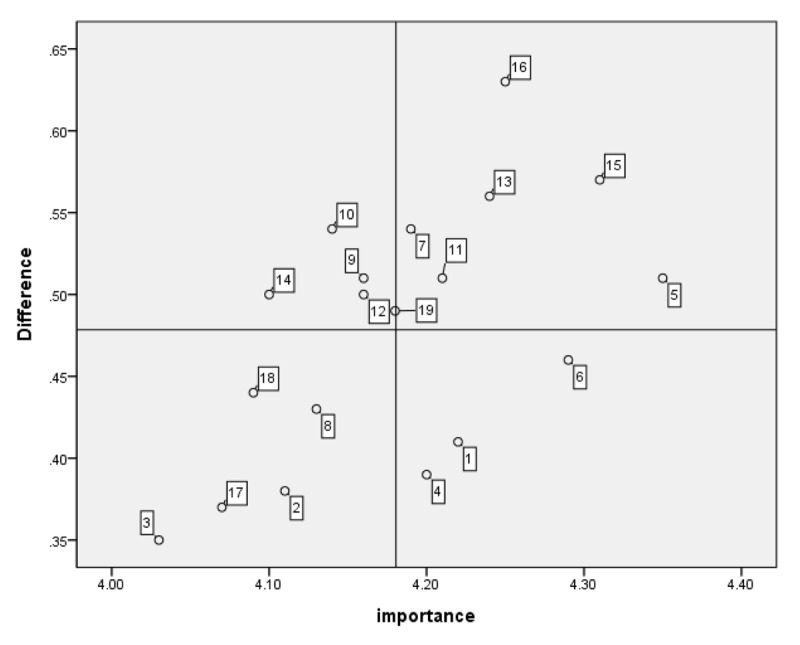
Educational needs of all integrated care professionals using the Locus for Focus Model. Cut-off value: Importance = 4.18, difference (importance-performance) = 0.48. Educational needs in Quadrant 1, such as no. 16 (utilizing ICT appropriately for integrated care), and no. 15 (collaboratively working with multidisciplinary professionals), were the educational needs of all integrated care professionals.

This study administered 19 items derived from the core competencies of integrated care to all the integrated care professionals. The average importance and performance scores for the competencies and educational needs are presented in Table 2. No. 5 (working collaboratively across integrated care settings to improve professionals’ and community residents’ experience of care) was the most important competency according to the respondents, with a score of 4.35 ± 0.77 (mean ± standard deviation), and was also the most-performed competency, with a score of 3.84 ± 0.99. The highest educational needs score was analyzed using the Borich Needs Assessment Model for Item 16 (utilizing information and communication technology [ICT] appropriately for integrated care), and the second highest was for Item 15 (collaboratively working with multidisciplinary professionals). However, according to the Locus for Focus Model, no. 16 appeared in the HL quadrant, whereas nos. 15 and 5 appeared in the HH quadrant (Figure 1).

**Table 2:** Educational needs of all integrated-care professionals.


ITEM NO. (N = 210)	IMPORTANCE		PERFORMANCE		IMPORTANCE MINUS PERFORMANCE	PAIRED *T*	*p*	BORICH SCORE	BORICH RANK	QUADRANT IN LOCUS FOR FOCUS MODEL
		
	MEAN ± SD	RANK	MEAN ± SD	RANK	MEAN ± SD					

1	4.22 ± 0.74	6	3.81 ± 0.84	3	0.41 ± 0.77	8.15	<.001	1.71	15	LH

2	4.11 ± 0.80	15	3.73 ± 0.87	6	0.38 ± 0.80	7.38	<.001	1.57	17	LH

3	4.03 ± 0.86	19	3.68 ± 0.92	11	0.35 ± 0.84	6.45	<.001	1.41	19	LL

4	4.20 ± 0.81	8	3.81 ± 0.88	4	0.39 ± 0.81	7.55	<.001	1.66	16	LH

5	4.35 ± 0.77	1	3.84 ± 0.99	1	0.51 ± 0.88	9.04	<.001	2.22	6	HH

6	4.29 ± 0.80	3	3.83 ± 0.93	2	0.46 ± 0.78	9.03	<.001	1.96	12	LH

7	4.19 ± 0.83	9	3.65 ± 0.99	15	0.54 ± 0.89	9.41	<.001	2.26	4	HL

8	4.13 ± 0.88	14	3.70 ± 1.01	9	0.43 ± 0.86	7.73	<.001	1.76	14	LL

9	4.16 ± 0.84	11	3.65 ± 0.99	14	0.51 ± 0.85	9.22	<.001	2.10	8	HL

10	4.14 ± 0.84	13	3.60 ± 1.01	19	0.54 ± 0.96	8.71	<.001	2.23	5	HL

11	4.21 ± 0.84	7	3.70 ± 0.99	7	0.51 ± 0.98	8.12	<.001	2.15	7	HL

12	4.16 ± 0.83	12	3.66 ± 0.95	13	0.50 ± 0.85	9.12	<.001	2.07	9	HL

13	4.24 ± 0.86	5	3.68 ± 1.04	12	0.56 ± 0.96	8.94	<.001	2.36	3	HL

14	4.10 ± 0.88	16	3.61 ± 0.96	18	0.50 ± 0.84	9.18	<.001	2.04	11	HL

15	4.31 ± 0.77	2	3.74 ± 1.02	5	0.57 ± 0.93	9.45	<.001	2.45	2	HH

16	4.25 ± 0.85	4	3.62 ± 1.09	17	0.63 ± 1.07	9.19	<.001	2.70	1	HL

17	4.07 ± 0.79	18	3.70 ± 0.93	8	0.37 ± 0.81	7.14	<.001	1.52	18	LL

18	4.09 ± 0.82	17	3.65 ± 0.94	15	0.44 ± 0.87	7.78	<.001	1.78	13	LL

19	4.18 ± 0.85	10	3.69 ± 1.00	10	0.49 ± 0.90	8.52	<.001	2.07	10	HL


HH: high discrepancy, high importance; HL: high discrepancy, low performance; LH: low discrepancy, high importance; LL: low discrepancy, high importance.

### Educational Needs in the Community-Care Competencies of Administrators

**Figure 2:**
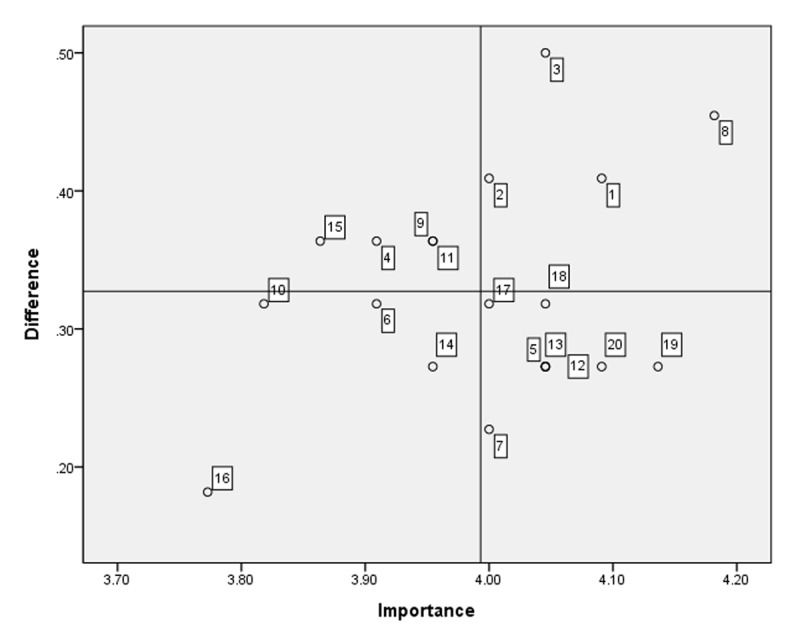
Educational needs of community-care administrators using the Locus for Focus Model. Cut-off value: Importance = 3.99, difference (importance-performance) = 0.33. Educational needs in Quadrant 1, such as no. 8 (building effective working relationships with other professionals working in public/private institutions) and no. 3 (developing policies relevant to integrated care based on circumstances and problems in the community), urgently address the educational needs of community-care administrators.

Twenty items derived from community-care administrator competencies in integrated care were administered to the community-care administrators. The average importance and performance scores for competencies and educational needs are presented in Table 3. Item 8 (building effective working relationships with other professionals working in public or private institutions) was the most important skill according to the respondents, with a score of 4.18 ± 0.80. Item 19 (planning finance, budget, and business, and monitoring the cost-effective use of finance and resources) was the most performed skill, with a score of 3.86 ± 0.99. The highest educational needs score analyzed using the Borich Needs Assessment Model was for no. 3 (developing policies relevant to integrated care based on circumstances and problems in the community), and the second highest was for no. 8. According to the Locus for Focus Model, no. 1 (identifying high-priority needs related to healthcare and social welfare in the community), no. 2 (identifying healthcare and social welfare problems in the community), and nos. 3 and 8 appeared in the HH quadrant (Figure 2).

**Table 3:** Educational needs for community-care administrators in integrated care.


ITEM NO. (N = 22)	IMPORTANCE		PERFORMANCE		IMPORTANCE MINUS PERFORMANCE	PAIRED *T*	P	BORICH SCORE	BORICH RANK	QUADRANT IN LOCUS FOR FOCUS MODEL
		
	MEAN ± SD	RANK	MEAN ± SD	RANK	MEAN ± SD					

1	4.09 ± 0.75	3	3.68 ± 1.09	9	0.41 ± 1.01	1.904	.071	1.67	3	HH

2	4.00 ± 0.82	10	3.59 ± 0.91	12	0.41 ± 0.80	2.409	.025	1.64	4	HH

3	4.05 ± 0.90	5	3.55 ± 0.91	17	0.50 ± 0.91	2.569	.018	2.02	1	HH

4	3.91 ± 0.92	16	3.55 ± 1.14	17	0.36 ± 0.79	2.160	.042	1.42	7	HL

5	4.05 ± 0.79	5	3.77 ± 0.92	3	0.27 ± 0.83	1.547	.137	1.10	15	LH

6	3.91 ± 0.92	16	3.59 ± 0.91	12	0.32 ± 0.65	2.309	.031	1.24	11	LL

7	4.00 ± 0.93	10	3.77 ± 1.07	3	0.23 ± 0.61	1.742	.096	0.91	19	LH

8	4.18 ± 0.80	1	3.73 ± 1.03	7	0.45 ± 0.86	2.485	.021	1.90	2	HH

9	3.95 ± 0.84	13	3.59 ± 0.91	12	0.36 ± 0.66	2.592	.017	1.44	5	HL

10	3.82 ± 0.96	19	3.50 ± 1.01	19	0.32 ± 0.65	2.309	.031	1.21	12	LL

11	3.95 ± 0.95	13	3.59 ± 0.96	12	0.36 ± 0.79	2.160	.042	1.44	5	HL

12	4.05 ± 0.90	5	3.77 ± 1.02	3	0.27 ± 0.63	2.027	.056	1.10	15	LH

13	4.05 ± 0.79	5	3.77 ± 0.92	3	0.27 ± 0.77	1.667	.110	1.10	15	LH

14	3.95 ± 0.84	13	3.68 ± 0.89	9	0.27 ± 0.63	2.027	.056	1.08	18	LL

15	3.86 ± 0.94	18	3.50 ± 1.19	19	0.36 ± 0.90	1.891	.073	1.40	8	HL

16	3.77 ± 0.97	20	3.59 ± 1.10	12	0.18 ± 0.66	1.283	.213	0.69	20	LL

17	4.00 ± 0.82	10	3.68 ± 0.95	9	0.32 ± 0.78	1.914	.069	1.27	10	LH

18	4.05 ± 0.79	5	3.73 ± 1.03	7	0.32 ± 0.78	1.914	.069	1.29	9	LH

19	4.14 ± 0.89	2	3.86 ± 0.99	1	0.27 ± 0.77	1.667	.110	1.13	13	LH

20	4.09 ± 0.97	3	3.82 ± 1.05	2	0.27 ± 0.77	1.667	.110	1.12	14	LH


HH: high discrepancy, high importance; HL: high discrepancy, low performance; LH: low discrepancy, high importance; LL: low discrepancy, high importance.

### Educational Needs in the Competencies of Care Coordinators

**Figure 3:**
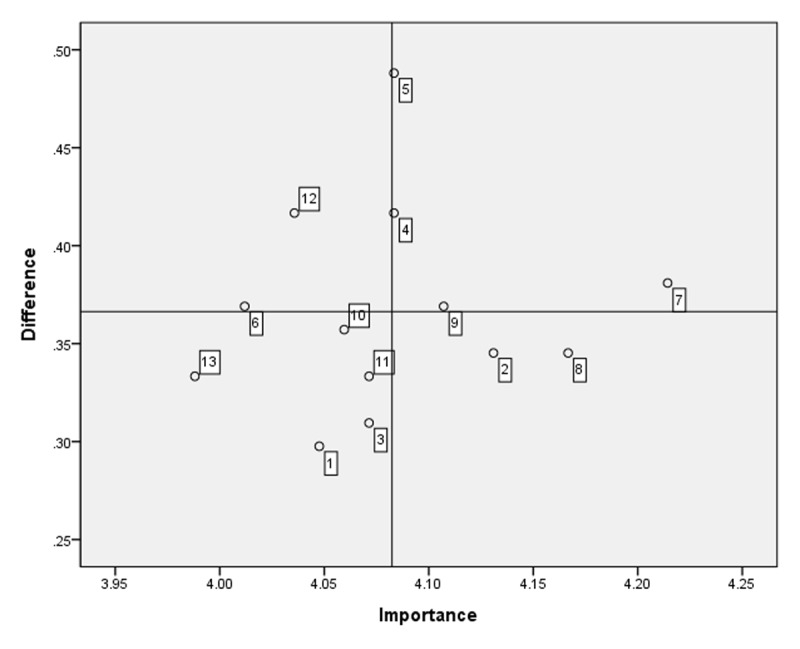
Educational needs of care coordinators using the Locus for Focus Model. Cut-off value: Importance = 4.08, difference (importance-performance) = 0.37. Educational needs in Quadrant 1, such as no. 7 (referring to community residents with health care and social welfare needs to adequate services), no. 4 (accurately determining patients’ comprehensive needs), and no. 5 (contributing to developing care plans to meet individuals’ health and social care needs), were the urgently addressed educational needs of community-care coordinators.

Thirteen items derived from community-care coordinator competencies in integrated care were administered to community-care coordinators. The average importance and performance scores for the competencies and educational needs are presented in Table 4. No. 7 (referring to community residents with health care and social welfare needs to adequate services) was the most important skill according to the respondents, with a score of 4.21 ± 0.79, and was the most-performed skill, with a score of 3.83 ± 0.92. The highest educational needs score analyzed using the Borich Needs Assessment Model was for no. 7, and the second highest was for no. 8 (identifying community residents’ satisfaction with received services). According to the Locus for Focus Model, no. 4 (determining patients’ comprehensive needs accurately), no. 5 (contributing to developing care plans to meet individuals’ health and social care needs), and nos. 7 and 9 (monitoring the quality of services to meet the quality standards and requirements) appeared in the HH quadrant (Figure 3).

**Table 4:** Educational needs for care coordinators in integrated care.


ITEM NO. (N = 84)	IMPORTANCE		PERFORMANCE		IMPORTANCE MINUS PERFORMANCE	PAIRED *T*	*P*	BORICH SCORE	BORICH RANK	QUADRANT IN LOCUS FOR FOCUS MODEL
		
	MEAN ± SD	RANK	MEAN ± SD	RANK	MEAN ± SD					

1	4.05 ± 0.89	8	3.75 ± 0.94	5	0.30	3.85	<.001	1.20	5	LL

2	4.13 ± 0.80	3	3.79 ± 0.95	3	0.35	3.96	<.001	1.43	3	LH

3	4.07 ± 0.83	6	3.76 ± 0.90	4	0.31	3.73	<.001	1.26	4	LL

4	4.08 ± 0.89	5	3.67 ± 0.94	9	0.42	5.11	<.001	1.70	9	HH

5	4.08 ± 0.81	5	3.60 ± 1.08	13	0.49	4.42	<.001	1.99	13	HH

6	4.01 ± 0.78	10	3.64 ± 0.93	11	0.37	3.87	<.001	1.48	11	HL

7	4.21 ± 0.79	1	3.83 ± 0.92	1	0.38	3.98	<.001	1.61	1	HH

8	4.17 ± 0.80	2	3.82 ± 0.85	2	0.35	4.62	<.001	1.44	2	LH

9	4.11 ± 0.82	4	3.74 ± 0.87	6	0.37	4.90	<.001	1.52	6	HH

10	4.06 ± 0.88	7	3.70 ± 0.95	8	0.36	3.94	<.001	1.45	8	LL

11	4.07 ± 0.74	6	3.74 ± 0.87	6	0.33	3.99	<.001	1.36	6	LL

12	4.04 ± 0.72	9	3.62 ± 0.89	12	0.42	5.47	<.001	1.68	12	HL

13	3.99 ± 0.81	11	3.65 ± 0.90	10	0.33	4.07	<.001	1.33	10	LL


### Educational Needs in the Competencies of Healthcare and Social Care Service Providers

**Figure 4:**
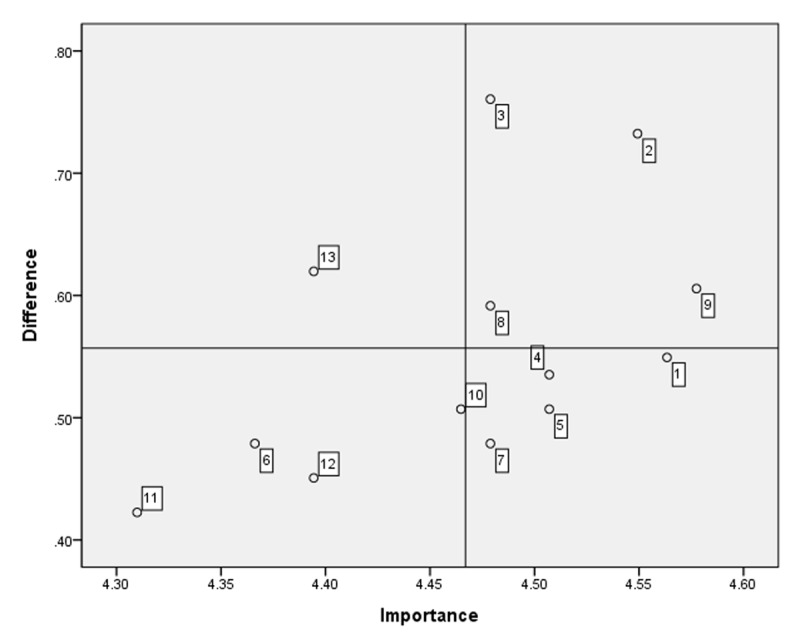
. Educational needs of healthcare and social care service providers using the Locus for Focus Model. Cut-off value: Importance = 4.47, difference (importance-performance) = 0.56. Educational needs in Quadrant 1, such as no. 3 (promoting local services effectively), no. 2 (planning local services based on the needs of community residents), and no. 9 (helping community residents confront emergencies/crises) were the urgently addressed educational needs of healthcare and social care service providers.

Thirteen items derived from community-care service providers’ competencies in integrated care were administered to community-care service providers. The average importance and performance scores for the competencies and educational needs are presented in Table 5. Item 9 (helping community residents confront emergencies or crises) was the most important competency according to the respondents, with a score of 4.58 ± 0.62. Item 1 (identifying the health and social care needs of community residents to provide person-centered services) was the most performed competency, with a score of 4.01 ± 0.84. The highest educational needs score analyzed using the Borich Needs Assessment Model was for no. 3 (promoting local services effectively), and the second highest was for no. 2 (planning local services based on the needs of community residents). According to the Locus for Focus Model, no. 2, no. 3, no. 8 (undertaking training and development of the workforce providing services), and no. 9 appeared in the HH quadrant (Figure 4).

**Table 5:** Educational needs for healthcare and social-care service providers in integrated care.


ITEM NO. (N = 71)	IMPORTANCE		PERFORMANCE		IMPORTANCE MINUS PERFORMANCE	PAIRED *T*	*P*	BORICH SCORE	BORICH RANK	QUADRANT IN LOCUS FOR FOCUS MODEL
		
	MEAN ± SD	RANK	MEAN ± SD	RANK	MEAN ± SD					

1	4.56 ± 0.63	2	4.01 ± 0.84	1	0.55 ± 0.79	5.87	<.001	2.51	6	LH

2	4.55 ± 0.65	3	3.82 ± 0.95	11	0.73 ± 1.04	5.93	<.001	3.33	2	HH

3	4.48 ± 0.73	6	3.72 ± 1.03	13	0.76 ± 1.08	5.96	<.001	3.41	1	HH

4	4.51 ± 0.71	4	3.97 ± 0.89	4	0.54 ± 0.77	5.85	<.001	2.41	7	LH

5	4.51 ± 0.67	4	4.00 ± 0.79	2	0.51 ± 0.77	5.53	<.001	2.29	8	LH

6	4.37 ± 0.81	12	3.89 ± 0.98	8	0.48 ± 0.94	4.30	<.001	2.09	11	LL

7	4.48 ± 0.71	6	4.00 ± 0.88	2	0.48 ± 0.83	4.89	<.001	2.14	10	LH

8	4.48 ± 0.65	6	3.89 ± 0.95	8	0.59 ± 1.02	4.88	<.001	2.65	5	HH

9	4.58 ± 0.62	1	3.97 ± 0.91	4	0.61 ± 0.80	6.37	<.001	2.77	3	HH

10	4.46 ± 0.67	9	3.96 ± 0.80	6	0.51 ± 0.75	5.67	<.001	2.26	9	LL

11	4.31 ± 0.77	13	3.89 ± 0.80	8	0.42 ± 0.84	4.24	<.001	1.82	13	LL

12	4.39 ± 0.69	10	3.94 ± 0.81	7	0.45 ± 0.81	4.71	<.001	1.98	12	LL

13	4.39 ± 0.69	10	3.77 ± 0.87	12	0.62 ± 0.80	6.53	<.001	2.72	4	HL


### Educational Needs in the Competencies of Community Health Champions

**Figure 5:**
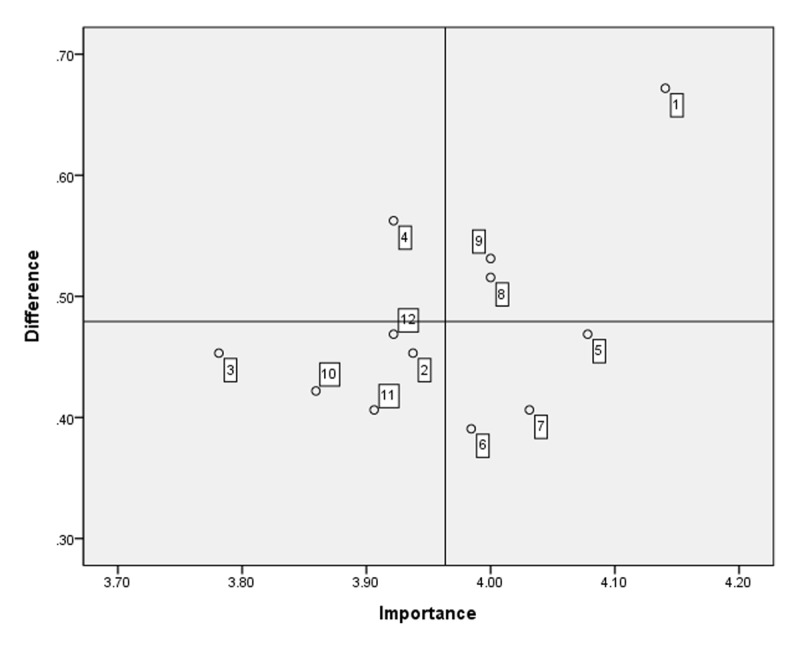
Educational needs of community health champions using the Locus for Focus Model. Cut-off value: Importance = 3.96, difference (importance-performance) = 0.48. Educational needs in Quadrant 1, such as no. 1 (identifying health and social care-related problems of community residents), no. 9 (cooperating with community-based organizations/institutions for integrated care), and no. 8 (playing a leading role when designing health- and social-related business plans for community residents), were the urgently addressed educational needs of community health champions.

Twelve items derived from the competencies of community health champions in integrated care were administered to community residents who worked as community health champions. The average importance and performance scores for the competencies and educational needs are presented in Table 6. Item 1 (identifying health and social care-related problems of community residents) was the most important competency according to the respondents, with a score of 4.14 ± 0.77. Item 7 (participating in decision-making relevant to health and social care needs) was the most performed competency, with a score of 3.63 ± 0.95. The highest educational needs score analyzed using the Borich Needs Assessment Model was for Item 1, and the second highest was for Item 4 (understanding the importance of participating in the integrated care process). According to the Locus for Focus Model, no. 1, no. 8 (playing a leading role when designing health- and social-related business plans for community residents), and no. 9 (cooperating with community-based organizations/institutions for integrated care) appeared in the HH quadrant (Figure 5).

**Table 6:** Educational needs for community health champions in integrated care.


ITEM NO. (N = 64)	IMPORTANCE		PERFORMANCE		IMPORTANCE MINUS PERFORMANCE	PAIRED *T*	*P*	BORICH SCORE	BORICH RANK	QUADRANT IN LOCUS FOR FOCUS MODEL
		
	MEAN ± SD	RANK	MEAN ± SD	RANK	MEAN ± SD					

1	4.14 ± 0.77	1	3.47 ± 0.94	7	0.67 ± 0.96	5.597	<.001	2.78	1	HH

2	3.94 ± 0.85	7	3.48 ± 0.94	5	0.45 ± 0.85	4.249	<.001	1.78	7	LL

3	3.78 ± 0.83	12	3.33 ± 0.91	12	0.45 ± 0.94	3.850	<.001	1.71	8	LL

4	3.92 ± 0.90	8	3.36 ± 0.98	11	0.56 ± 0.97	4.621	<.001	2.21	2	HL

5	4.08 ± 0.88	2	3.61 ± 1.00	2	0.47 ± 1.01	3.722	<.001	1.91	5	LH

6	3.98 ± 0.85	6	3.59 ± 0.97	3	0.39 ± 0.92	3.400	.001	1.56	12	LH

7	4.03 ± 0.84	3	3.63 ± 0.95	1	0.41 ± 1.03	3.141	.003	1.64	9	LH

8	4.00 ± 0.87	4	3.48 ± 0.98	5	0.52 ± 1.02	4.031	<.001	2.06	4	HH

9	4.00 ± 0.84	4	3.47 ± 0.89	7	0.53 ± 0.94	4.510	<.001	2.13	3	HH

10	3.86 ± 0.91	11	3.44 ± 0.97	10	0.42 ± 0.92	3.659	.001	1.63	10	LL

11	3.91 ± 0.83	10	3.50 ± 0.89	4	0.41 ± 0.89	3.669	.001	1.59	11	LL

12	3.92 ± 0.86	8	3.45 ± 0.92	9	0.47 ± 0.82	4.596	<.001	1.84	6	LL


## Discussion

This study identified differences between integrated care professionals’ perceptions of importance and their performance regarding integrated care competency and presented educational needs in a matrix.

A core competency of educational needs highly perceived by integrated care professionals was “utilizing ICT and digital technology to provide integrated care.” Integrated care delivery can be effectively supported by ICT, such as e-health [35], which enhances opportunities for remote digital data sharing, communication, and consultation to improve integrated care [36]. Previous research has established that core competencies in digitalization from a healthcare viewpoint include knowledge of digital technology and digital skills [37]. Furthermore, the digitalization of care delivery may require changes in practice and workflow. Practical training programs for digital technology, such as patient link systems, e-health, and telehealth, should be included in the integrated care curriculum to cope with the changing trends in care delivery processes.

Another highly perceived educational need for core competency was “cooperating with multidisciplinary groups.” Each integrated care professional learns the principles of clinical practice guidelines in their sector, such as nursing, public health, social services, and public administration. However, professionals’ understanding of interprofessional practice may differ since IPE is not a standard part of all disciplines. Active learning environments such as workshops provide fertile grounds for nurturing interprofessional understanding and building a collaborative spirit [38]. Before tackling real-world integrated care in communities, professionals require training in core skills such as seamless collaboration across disciplines, building trust and rapport with residents, and constructively resolving disagreements with stakeholders. Simulation-based IPE needs to be considered as a versatile platform for healthcare professionals to practice interprofessional collaboration in realistic scenarios [39,40].

Administrators with high educational needs are developing policies relevant to integrated care based on circumstances and problems in the community and identifying high-priority needs and problems in the community. Integrated care administrators must play a critical role in strategic planning and execution, resource management, and performance evaluation. A previous study reported that a critical challenge for healthcare managers lies in designing policies, procedures, and practices that effectively integrate care across specialized units and are implemented within the existing social dynamics and operational processes of each unit [41]. By tailoring integrated care policies to address specific situations and emerging issues as they arise and pinpointing the most pressing issues and unmet needs within the community, it is possible to demonstrate strong leadership in integrated care.

The competencies “referring to community residents with healthcare and social-welfare needs to adequate services,” “determining the patients’ comprehensive needs accurately,” and “contributing to developing care plans to meet individuals’ health and social care needs” indicated that high educational needs on care coordinators. This result is consistent with a previous study’s result that care coordinators noted needs-assessment training should be emphasized to identify and understand patients’ specific needs and focus on essential program features and procedures [42]. Strengthening the findings of recent research, this study underscores the critical role of providing care coordinators with comprehensive training that encompasses diverse aspects of patient care [43].

The high educational needs of health and social care providers include promoting local services effectively, planning local services based on the needs of community residents, and undertaking training and development of the workforce that provides services. These results can be explained by the development of skills and knowledge relevant to both one’s external professional environment and the internal professional development of health and social care providers. Health and social care providers must establish good inter-organizational relationships [44] and ongoing consultation with community stakeholders [45] as an effective approach to integrated care. Previous studies have also reported that a lack of employment and training for integrated care providers could be a significant barrier [46]. For health and social care providers, the educational program needs to focus on strategies to improve the quality of services and human resources and establish strong external relationships and a positive image to provide integrated care services effectively.

The high educational needs score identified health and social care-related problems of community residents and understood the importance of participating in the integrated care process. These results are similar to those of previous studies reporting that delivering peer support programs presents various challenges, including matching peers, setting boundaries, and earning professional trust [47]. Although many studies have investigated peer support for specific diseases, such as mental health or survival, in clinical settings, peer programs offered in community settings can also be a valuable source of support and guidance [47] because shared experiences in wellness ignite support and hope, empowering recovery for those managing behavioral health conditions [48,49]. To facilitate health champions to play a leading role in integrated care, appropriate training programs for health champions should also be implemented.

Multi-stakeholder engagement and trust building through collaborative governance are key to integrating care work [50]. This approach often uses inter-organizational networks or partnerships between healthcare providers and professionals [51]. In a previous study, a network platform for integrated care professionals actively influenced their interactions, allowing diverse and even conflicting goals to coexist and potentially be achieved simultaneously [52]. In this regard, designing and implementing educational programs is a promising strategy for governing and facilitating network building among these professionals, ultimately offering new ways for multidisciplinary cooperation by helping them acquire knowledge to implement the governance of integrated care [53], and organizing and strengthening relevant systems and governance [54]. This study is the first step toward identifying the educational needs of integrated care professionals to develop better education programs in the future.

### Strengths and Limitations

The need for integrated care is increasing owing to the aging population. The increasing demand for person-centered care for the elderly necessitates integrated medical, nursing, and social service models. Working together can provide comprehensive and coordinated care tailored to each individual’s unique needs and preferences. This study investigated the educational needs of integrated care professionals based on their competencies. This further enables evidence-based education program designs and allows learners to apply their knowledge practically. The study also included a core professional group comprising community health champions who needed to be included in integrated care in community settings. This would provide an opportunity to train peer supporters, who are also community residents, as experts to facilitate public participation in the integrated care process. However, this study had several limitations. First, it was conducted in one country, and specific cultural or political characteristics may have affected the results. Future studies should include multiple countries to compare these differences. Selection bias may have affected the validity and generalizability of our findings to a broader population. The sample was drawn from integrated care professionals in several cities in South Korea who agreed to participate in this study. Moreover, the number of participants in the community-care administrator group was relatively small because they were public officers working in public centers.

## Conclusion

This study demonstrates the educational needs of integrated care professionals in community settings. Each professional has a specific educational need to implement integrated care in their practice; moreover, they want better interprofessional cooperation through networking and collaborative strategies. The results of this study may be utilized as fundamental data by future instructors to provide evidence-based education programs with the potential to improve integrated care by increasing the competency levels of professionals. In future studies, integrated care curriculum development should consider the priorities of the professionals’ preferences and challenges based on their educational needs.

## Additional File

The additional file for this article can be found as follows:

10.5334/ijic.7674.s1 Appendix 1. The competency items of questionnaire.
