# Supplementary material for: Interprofessional Educational Needs for Shared Governance of Integrated Care
Source: Int J Integr Care. 2024 May 6;24(1):15. doi: 10.5334/ijic.7674 (PMC11086590; doi:10.5334/ijic.7674)
Supplement: Appendix 1. — The competency items of questionnaire. [file ijic-24-2-7674-s1.pdf]

Appendix 1. The competency items of questionnaire

Part 1. Core integrated-care competencies

1. Understanding the definition and concepts of integrated care.
2. Understanding timely conditions and policies relevant to integrated care .
3. Understanding the difference between traditional health care policy and integrated care policy.
4. Understanding person-centered integrated care.
5. Working collaboratively across integrated care settings to improve professionals' and community residents' experience of care.
6. Effectively communicating with community residents and other professionals.
7. Demonstrating inclusive leadership and professional accountability.
8. Shared decision making with community residents.
9. Diagnosing health and social problems and finding solutions based on community residents' needs.
10. Building relationships or networking with community residents and other professionals.
11. Effectively communicating community residents' needs within a multidisciplinary team.
12. Understanding the social value of integrated care contributing to the community.
13. Establishing community resident-centered integrated health and social-care delivery system.
14. Sharing information related to integrated care with other professionals and community residents.
15. Collaboratively working with multidisciplinary professionals .
16. Using ICT (Information and Communication Technology) appropriately for integrated care.
17. Cooperating or participating in research relevant to integrated care.
18. Improving public perceptions of integrated care professionals.
19. Motivating community residents to participate in the process of integrated care.

Part 2. Community-care competencies of administrators

1. Identifying high-priority needs related to healthcare and social welfare in the community.
2. Identifying healthcare and social welfare-related problems in the community.
3. Developing policies relevant to integrated care based on circumstances and problems in the community.
4. Selecting potential recipients of integrated care services based on community diagnosis.
5. Planning integrated care strategies locally.
6. Implementing integrated care planning.
7. Investing in and securing local resources for living in place in the community.
8. Building effective working relationships with other professionals working in public/private

institutions.

9. Effectively chairing or leading multidisciplinary team meetings.
10. Motivating community residents to organize community gatherings to achieve the goals of integrated care.
11. Finding solutions based on the ideas and opinions of community residents.
12. Coordinating individuals within multidisciplinary teams to sustain clarity of roles and accountability.
13. Ensuring a cooperative environment for multidisciplinary team members.
14. Contributing to the delivery of supervision of other integrated care professionals.
15. Educating other staff and colleagues based on their competence.
16. Considering the voice of the community, speaking with one member acting as a representative.
17. Establishing appropriate local systems for ensuring high quality of care delivery system.
18. Distributing local resources effectively based on clients' needs.
19. Planning finance, budget, and business and monitoring cost-effectiveness of the use of finance and resources.
20. Evaluating the project results related to integrated care to shape ongoing improvement.

Part 3. Competencies of care coordinators

1. Discovering community residents who might need integrated care from information linkage system.
2. Identifying community residents who might benefit from integrated care services based on initial assessment.
3. Assessing comprehensive health and social needs of community residents.
4. Determining the patients' comprehensive needs accurately.
5. Contributing to developing care plans to meet individuals' health and social care needs.
6. Referring community residents to multidisciplinary meetings to make care plans.
7. Referring community residents with health care and social welfare needs to adequate services.
8. Identifying community residents' satisfaction with received services.
9. Monitoring the quality of services to meet the quality standards and requirements.
10. Modifying established care plans, implementing continued care management based on the community residents' situation.
11. Cooperating with community-based organizations and local institutions for integrated care.
12. Instructing community residents with long-term conditions on how to care for themselves.
13. Improving the work process based on integrated care performance results.

Part 4. Competencies of healthcare and social-care service providers

1. Identifying health and social care needs of community residents to provide person-centered services.
2. Planning local services based on the needs of community residents.
3. Promoting local services effectively.
4. Developing cooperative relationship with integrated care-related stakeholders (e.g., professionals, community residents).
5. Sharing resources and information with relevant organizations to benefit community residents.
6. Evaluating the community residents' experiences and changes after receiving services.
7. Improving and designing new local services.
8. Undertaking training and development of the workforce providing services.
9. Helping the client confront emergencies/crises.
10. Identifying and prioritizing community residents' complex health and social care needs and arranging adequate services based on the types of clients (e.g., older people, disabled people, women, children, the mentally ill).
11. Educating and monitoring community residents while practicing self-care.
12. Identifying the primary community residents' health and social care needs and providing appropriate service.
13. Consulting with community residents to keep them motivated

Part 5. Competencies of community health champions

1. Identifying health- and social-care related problems of community residents.
2. Identifying the causes of health- and social-related problems of community residents.
3. Searching for health- and social-related information and services.
4. Understanding the importance of participating in the integrated care process.
5. Encouraging other community residents to participate in community activities, including community gatherings.
6. Constructing community-leading organizations on integrated care.
7. Participating in decision making relevant to health- and social-care needs.
8. Playing a leading role when designing health- and social-related business plans for community residents.
9. Cooperating with community-based organizations/institutions for integrated care.
10. Developing self-help groups and actively participating in their meetings.
11. Participating in evaluating performance results of integrated care and suggesting improvements.
12. Contributing to, and participating in, research relevant to integrated care.
